# Alteration of Pituitary Tumor Transforming Gene-1 Regulates Trophoblast Invasion via the Integrin/Rho-Family Signaling Pathway

**DOI:** 10.1371/journal.pone.0149371

**Published:** 2016-02-22

**Authors:** Seung Mook Lim, Hee Yeon Jang, Ji Eun Lee, Joong Sik Shin, Sun-Hwa Park, Bo Hyun Yoon, Gi Jin Kim

**Affiliations:** 1 Department of Biomedical Science, CHA University, Seongnam-si, 463-400, Republic of Korea; 2 Department of Obstetrics and Gynecology, College of Medicine, CHA University, Seoul, Republic of Korea; 3 Institute of Human Genetics, Department of Anatomy, Korea University College of Medicine, Seoul, Republic of Korea; 4 Department of Obstetrics and Gynecology, Seoul National University College of Medicine, Seoul, Republic of Korea; China Medical University, TAIWAN

## Abstract

Trophoblast invasion ability is an important factor in early implantation and placental development. Recently, pituitary tumor transforming gene 1 (PTTG1) was shown to be involved in invasion and proliferation of cancer. However, the role of PTTG1 in trophoblast invasion remains unknown. Thus, in this study we analyzed PTTG1 expression in trophoblasts and its effect on trophoblast invasion activity and determined the mechanism through which PTTG1 regulates trophoblast invasion. Trophoblast proliferation and invasion abilities, regardless of PTTG1 expression, were analyzed by quantitative real-time polymerase chain reaction, fluorescence-activated cell sorting analysis, invasion assay, western blot, and zymography after treatment with small interfering RNA against PTTG1 (siPTTG1). Additionally, integrin/Rho-family signaling in trophoblasts by PTTG1 alteration was analyzed. Furthermore, the effect of PTTG1 on trophoblast invasion was evaluated by microRNA (miRNA) mimic and inhibitor treatment. Trophoblast invasion was significantly reduced through decreased matrix metalloproteinase (MMP)-2 and MMP-9 expression when PTTG1 expression was inhibited by siPTTG1 (*p <* 0.05). Furthermore, knockdown of PTTG1 increased expression of integrin alpha 4 (ITGA4), ITGA5, and integrin beta 1 (ITGB1); otherwise, RhoA expression was significantly decreased (*p <* 0.05). Treatment of miRNA-186-5p mimic and inhibitor controlled trophoblast invasion ability by altering PTTG1 and MMP expression. PTTG1 can control trophoblast invasion ability via regulation of MMP expression through integrin/Rho-family signaling. In addition, PTTG1 expression and its function were regulated by miRNA-186-5p. These results help in understanding the mechanism through which PTTG1 regulates trophoblast invasion and thereby implantation and placental development.

## Introduction

The placenta, which is a temporary organ during pregnancy, is formed from the outer layer of the blastocyst (e.g., trophectoderm) [1]. The normal placenta plays a crucial role in fetal development by producing a variety of pregnancy-associated hormones and growth factors and supplying nutrients, and it acts as a maternal—fetal interface organ transporting waste products and gases [2]. Thus, normal placental development is important to maintain pregnancy as well as for fetal development. Particularly, trophoblasts originating from the trophectoderm of the blastocyst are the major cells found in the placenta, and their main function is invasion of the maternal uterine wall during early pregnancy via transformation of spiral arteries and differentiation of cytotrophoblasts (CTBs) into syncytiotrophoblasts [3, 4]. Several factors including environmental factors (e.g., hypoxia) and various cytokines strictly regulate trophoblast invasion activity [5, 6]. In previous reports, we demonstrated that hypoxia induced trophoblast invasion through dynamic alterations of integrin and matrix metalloproteinase (MMP) expression, especially through the down-regulation of integrin alpha 4 (ITGA4), which is observed in short-term hypoxia [7]. Additionally, trophoblast invasion can be modulated by the expression of various genes, such as adhesion molecules, MMPs, small guanosine triphosphatases (GTPases), epithelial—mesenchymal transition (EMT)-related factors, and microRNAs (miRNAs) [8–10].

Small non-coding miRNAs regulate target gene expression through post-transcriptional repression or cleavage of the target gene by matching the target mRNA 3’ untranslated region (UTR) [11]. Thus, miRNA is involved in various cellular processes, including proliferation, differentiation, invasion, and migration. In a previous report, Li et al demonstrated that miRNA-495 and miRNA-551a inhibited invasion and migration in gastric cancer by targeting the phosphatase of regenerating liver-3 oncogene [12]. Although the effects of trophoblasts on survival, invasion, and migration was recently reported to be regulated by miRNA-378a-5p [99], the correlation between trophoblast functions and diagnostic markers should be evaluated. Generally, factors capable of regulating trophoblast invasion are closely connected to cancer metastasis [13, 14]. A common characteristic of trophoblasts and tumors is their invasiveness [15, 16]. Although the precise mechanisms are unclear, those controlling invasion differ between trophoblasts and tumors. For example, trophoblast invasion reaches only one-third of the maternal uterine wall, whereas tumors invade uncontrollably.

Pituitary tumor transforming gene-1 (PTTG1), later identified as human securin involved in the control of chromosome segregation during the metaphase—anaphase transition in mitosis, is a proto-oncogene implicated in the progression of multiple cancer cell types through enhanced cancer proliferation [17, 18]. Additionally, PTTG1 is highly involved in many cellular processes, such as cell cycling, growth, DNA repair, and even senescence by regulating cell cycle—related proteins [19, 20]. Recently, Maik and Kakar demonstrated that PTTG1 is directly involved in the invasion of lung cancer cells by inducing EMT (epithelial-mesenchymal transition) via integrin-focal adhesion kinase signaling and alteration of MMPs [21]. However, whether PTTG1 can regulate the invasion and proliferation abilities of trophoblasts remains unclear.

Therefore, in the present study, we analyzed the expression of PTTG1 in human trophoblasts, including primary trophoblasts and the HTR-8/SVneo trophoblast cell line, which was established by transfection with simian virus 40 (SV40) large T antigen (Tag) in parental HTR-8, a trophoblast cell isolated from the first trimester of placental tissues. We also investigated the effect of PTTG1 expression on the invasion of trophoblasts using small interfering RNA (siRNA), and validated PTTG1 function using a miRNA target to PTTG1.

## Materials and Methods

### Placenta collection

The collection of human placental tissues and their use for research purposes was approved by the Institutional Review Board of CHA General Hospital, Seoul, Korea. All participants provided written, informed consent prior to sample collection. Normal placentas were collected from women who did not have any medical, obstetrical, or surgical complications, and who delivered at term (≥37 gestational weeks).

### Cell line and culture

Human primary trophoblast cells (PTB) were isolated from normal-term placenta. Briefly, placental villi tissues were chopped and washed in cold HBSS (Gibco, Rockville, MD, USA), and then centrifuged at room temperature for 5 min at 1,000 rpm. The tissue pellet was incubated with enzyme solution supplemented with 40 mg/mL of trypsin (Gibco), 20 mg/mL of DNase I (Gibco), and 24 U/mL dispase (Gibco) for 20 min at room temperature. The suspended supernatant was centrifuged at room temperature for 5 min at 1,000 rpm. The cell pellets were washed with culture medium supplemented with 1% penicillin/streptomycin (Gibco), 10% fetal bovine serum (FBS; Gibco), 2 mM L-glutamine (Gibco), 100 μM β-mercaptoethanol (Gibco), 1 μg/mL Heparin (Sigma, St. Louis, MO, USA), and 25 ng/mL fibroblast growth factor 4 (FGF4; Peprotech, Rocky Hill, NJ, USA), and then incubated in an atmosphere containing 5% CO_2_ at 37°C. The HTR-8/SVneo cell line, established from first-trimester cytotrophoblasts (CTBs) with the SV40 large T antigen, was provided by Dr. Charles H. Graham (Queen’s University, Kingston, Ontario, Canada) [22]. HTR-8/SVneo cells were cultivated in Roswell Park Memorial Institute 1640 medium (RPMI-1640) (Gibco) supplemented with 5% FBS (Gibco) and 100 U/mL penicillin/streptomycin (Gibco) at 37°C in an incubator with a humidified atmosphere of 5% CO_2_.

### Small interfering RNA (siRNA) and micro RNA (miRNA)

We used siRNA to analyze the effect of PTTG1 expression in primary trophoblast cells and first trimester CTBs HTR-8/SVneo, and the effect of ITGA4, MMP-2 and MMP-9 expression on HTR-8/SVneo cells. Briefly, the cells were harvested at 70–80% confluence and 1 × 10^6^ cells were seeded onto 100-mm culture dishes with serum-free medium (Opti-MEM; Gibco). Next, the cells were transiently transfected with 10 nM siRNA-PTTG1 (siPTTG1; Invitrogen, Carlsbad, CA, USA), 1 nM siRNA-ITGA4 (siITGA4; Santa Cruz Biotechnology, Inc., Santa Cruz, CA, USA), or 50nM MMP-2 and MMP-9 (siMMP-2, siMMP-9; Genolution, Seoul, Korea) using Lipofectamine 2000 (Invitrogen) and incubated for 48 h. In addition, HTR-8/SVneo cells were transfected with 30 nM miRNA-186-5p mimic and inhibitor (miR-186-5p; Genolution, Seoul, Korea) targeting PTTG1 expression or a Scramble control for 24 h. All transfection experiments were optimized for high efficiency and minimal cell damage. Transfected cells were harvested and stored at –20°C until future use.

### Rho inhibitor–*Clostridium botulinum* C3 transferase (C3 exoenzyme) treatment

To analyze the effects of the Rho ABC family on HTR-8/SVneo cells, 1 × 10^6^ cells were cultured onto 100-mm dishes and treated with or without 1 μg/mL of the Rho ABC inhibitor, C3 exoenzyme (Cytoskeleton, Inc., Denver, CO, USA), in serum-free culture medium supplemented with penicillin/streptomycin for 3 h. Next, the culture medium was replaced with one supplemented with 5% FBS and penicillin/streptomycin for 24 h, and then the cells were harvested for western blot analysis.

### MTT assay

Effect of PTTG1 and miR-186-5p on proliferation of HTR-8/SVneo cells was evaluated by the 3-(4,5-dimethylyhiazol-2-yl)-2,5-diphenyl tetrazollum bromide (MTT) assay. HTR-8/SVneo cells were seeded onto 96-well plate at density of 2.0 x 10^3^ with serum-free medium (Opti-MEM; Gibco). And then, the cells were transfected with 10nM siPTTG1 for 48 h or 30nM miR-186-5p for 24 h and cultivated at 37°C in an incubator with a humidified atmosphere of 5% CO_2_. After transfection, 0.5mg/ml MTT (Sigma) was added to each well, and the plate were placed in a 37°C incubator for 3 h. and then, dimethyl sulfoxide (DMSO, Sigma) was added for 10 min. The OD value at 570 nm was evaluated by a micro reader (BioTek, VT, USA). All experiments were performed triplicate.

### Fluorescence-activated cell sorting analysis

For cell cycle analysis, 1 × 10^6^ HTR-8/SVneo cells were seeded onto 100-mm culture dishes with serum-free medium (Opti-MEM; Gibco). The cells were transfected with 10 nM siPTTG1 for 48 h. The cells were fixed with ice-cold 70% ethanol for 10 min at room temperature, rehydrated in phosphate-buffered saline (PBS; Sigma), and collected by centrifugation at 2,000 rpm for 10 min. Next, the cells were resuspended with 10 μg/mL RNase A (Sigma) in PBS, incubated at 37°C for 30 min, and then stained with 50 μg/μL propidium iodide (PI; Sigma) to identify nonviable cells. Finally, 1 × 10^6^ cells were acquired and analyzed using a fluorescence-activated cell sorting (FACS) flow cytometer with CellQuest software (FACScan; BD Biosciences, Heidelberg, Germany). All experiments were performed in duplicate.

### Quantitative real-time polymerase chain reaction analysis

Total RNA was isolated from the cells and placental tissues using Trizol reagent (Invitrogen). cDNA was synthesized using Superscript III RNase H reverse transcriptase (Invitrogen) or a miR-X miRNA first-strand synthesis kit (Clontech Laboratories, Inc., Mountain View, CA, USA) according to the manufacturer’s protocol. The mRNA levels of the target genes were determined by quantitative real-time polymerase chain reaction (qRT-PCR) using SYBR Green master mix (Rox; Hoffmann-La Roche, Basel, Switzerland) or a mir-X miRNA qRT-PCR SYBR kit (Clontech Laboratories, Inc.) according to the manufacturer’s protocol. The specific primer sequences used for *PTTG1*, *ITGA4*, *MMP-9*, *MMP-2*, and mature *hsa-miR-186-5p* are shown in Table 1 (Table 1). The mRNA amplification conditions were pre-cooling at 95°C for 5 min, followed by 40 cycles at 95°C for 5 sec and 60°C for 30 sec. The miRNA amplification conditions were 95°C for 10 sec, followed by 40 cycles at 95°C for 5 sec and 60°C for 20 sec, with a final step at 95°C for 60 sec, 55°C for 30 sec, and 95°C for 30 sec. All experiments were performed in duplicate. Relative expression levels were calculated using the ΔΔCt method after normalization to β-actin or U6 as an internal control.

**Table 1:** Sequence of primers used in the study.

Gene	Primer sequence
PTTG1	F: 5’-AAG GAA AAT GGA GAA CA GGC-3’
	R: 5’-GCT TGG CTG TTT TTG TTT GAG G-3’
Integrin alpha4	F: 5’-CAG ACG TGC GAA CAG CAG CTC CAG-3’
	R: 5’-GCC AGT CCA GTA AGA TGA TCC TGG-3’
MMP-9	F: 5’-GAC GCA GAC ATC GTC ATC CAG TTT-3’
	R: 5’-GCC GCG CCA TCT GCG TTT-3’
MMP-2	F: 5’-CGG CCG CAG TGA CGG AAA-3’
	R: 5’CAT CCT GGG ACA GAC GGA AG-3’
miR-186-5p	F: 5’- CAA AGA ATT C TC CTT TGG GCT 3’

### Western blot analysis

Cells and placental tissues were homogenized, and then lysed in cold RIPA buffer (Sigma) with complete protease inhibitor cocktail (mini tablet; Roche, Mannheim, Germany) and phosphatase inhibitor cocktail II (A.G. Scientific Inc., San Diego, CA, USA). The protein concentrations of the collected lysates were determined using a bovine serum albumin (BSA) protein assay kit (Sigma) with a micro-plate reader (Molecular Devices, Sunnyvale, CA, USA). The protein lysates were denatured in 5× protein loading dye [50 mM-Tris HCl (pH 6.8), 2% sodium dodecyl sulfate (SDS), 1% 2-mercaptoethanol, 12.5 mM ethylenediaminetetraacetic acid, and 0.02% bromophenol blue] and loaded onto 8–15% SDS polyacrylamide gels (5~70 kDa = 15%, 20~100 kDa = 12%, 25~130 kDa = 10%, 35~300 kDa = 8%). The separated proteins were transferred onto a polyvinylidene difluoride membrane (Bio-Rad Laboratories, Inc., Berkeley, CA, USA). The membranes were blocked with 8% skim milk or 5% BSA (AMRESCO, Solon, OH, USA) for 1 h at room temperature and incubated with specific primary antibodies overnight at 4°C. S1 Table lists the primary antibodies used in the study (S1 Table). Membranes were washed and incubated with horseradish peroxidase—conjugated anti-rabbit immunoglobulin G (IgG; 1:25,000) or anti-mouse IgG (1:25,000) secondary antibodies for 1 h at room temperature, then detected using an electrochemiluminescence advance western blot detection kit (Amersham Biosciences, Uppsala, Sweden). The intensity of the gel bands was measured using ImageJ software (http://rsb.info.nih.gov/ij/, NIH, Bethesda, MD, USA). All experiments excluding integrin alpha4 (siITGA4), Rho A/B/C (C3 exoenzyme), and PTTG1 (microRNA-186) were performed in triplicate.

### Invasion assay using Transwell

Trophoblast invasion ability was determined using a 24-well cell culture insert system (Falcon, 8-μm pore size; BD Biosciences, Franklin Lakes, NJ, USA). Trophoblastic cells (2.5–4.0 × 10^4^) were loaded in the upper chamber with 0.3 mL serum-free medium, and culture medium containing 5% FBS was added to the lower chamber. Next, the cells were treated with small RNAs (siRNA, miRNA) using Lipofectamine 2000 (Invitrogen) to inhibit PTTG1, and ITGA4 expression and an inhibitor of the Rho ABC family, and incubated at 37°C for 24–48 h. The non-invading cells on the upper chamber were completely removed using cotton swabs, and invading cells on the lower surface were fixed with absolute methanol for 20 min and stained with Mayer’s hematoxylin (Dako, Carpinteria, CA, USA) at 37°C for 8 min. The number of stained cells was counted in at least seven randomly selected non-overlapping fields under light microscopy. All experiments were performed in triplicate.

### Zymography

The expression of MMPs, including MMP-2 and MMP-9, were determined by gelatin zymography. Supernatants from the trophoblastic cells were collected and separated onto 12% SDS polyacrylamide gels with 1 mg/mL gelatin. The separated gels were then washed twice for 40 min with renaturation buffer (Bio-Rad Laboratories, Inc.) and incubated overnight at 37°C in developing buffer containing 50 mM Tris-HCl (pH 7.4), 0.2 M NaCl, 5 mM CaCl_2_, and 1% Triton X-100. The next day, gels were stained with 10% acetic acid/40% methanol containing 0.5% Coomassie brilliant blue R-250 (Sigma) for 3 h and destained with destaining buffer containing 10% acetic acid/40% methanol. The density of unstained bands was used to detect enzyme expression. The intensity of gel bands was measured using the ImageJ processing program. All experiments were performed in triplicate.

### Double immunofluorescence

To analyze Cytokeratin 7 (Dako) and PTTG1 (Invitrogen) expression in placenta, placental tissues were embedded in OCT compound (Tokyo, Japan, Sakura Finetechnical Co. Ltd), and preserved at -80°C. Two serial sections of 6-μm thick were made of the same tissue specimen, and then fixed in 100% methanol. One section was stained with Hematoxylin and Eosin (H%E) solution. Another section was incubated in protein blocking solution (Dako) at room temperature for 30 min, and then incubated overnight at 4°C with mouse anti-cytokeratin7 (1:100 dilution; Dako) and rabbit anti-PTTG1 (1:100 dilution; Invitrogen), followed by 1 h of incubation at room temperature with Alexa 594-conjugated (1:200 dilution; Invitrogen) and Alexa 488–conjugated secondary antibody (1:200 dilution; Invitrogen). DAPI (Vector Laboratories, Burlingame, CA, USA) was used as a counterstain. Images were analyzed using an EVOS fl microscope (AMG, Mill Creek, WA, USA).

### Statistical analysis

Results are presented as mean ± standard error. Comparisons between two groups were performed using Student’s *t*-test, whereas comparisons among three or more groups using analysis of ANOVA on SAS software (SAS institute, NC, USA). * *p <* 0.05 and ** *p <* 0.001 were considered statistically significant.

## Results

### Expression of PTTG1 in trophoblast and placenta

**Fig 1:**
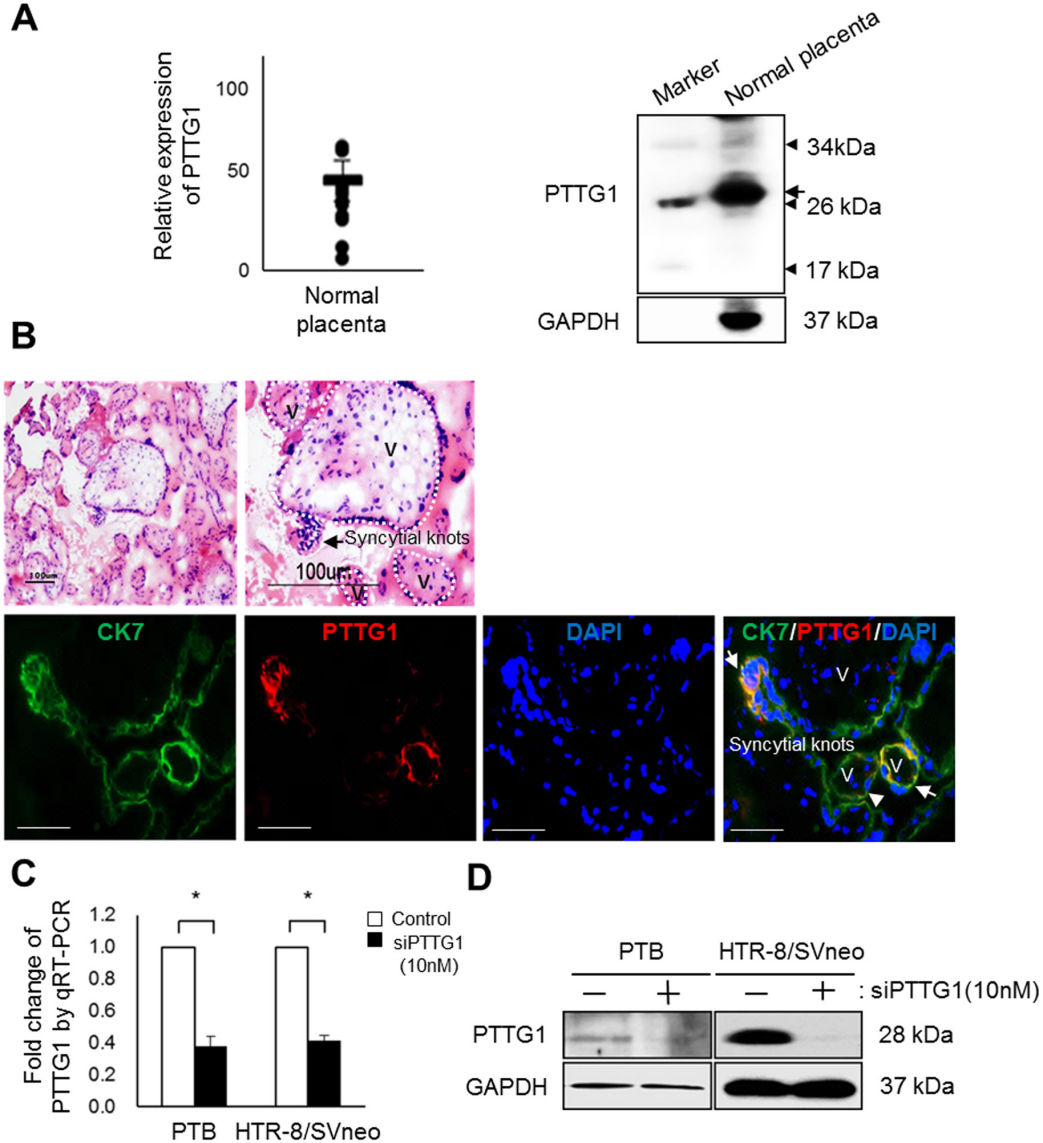
mRNA and PTTG1 protein expression in placenta and trophoblasts. (A) Expression of mRNA and PTTG1 protein in normal-term placenta (n = 14) was analyzed by qRT-PCR (left) and western blot (right). (B) Histology and localization of PTTG1 in normal-term placenta, as observed using H&E staining (upper) and double immunofluorescence (lower), respectively. Histological sections were stained with mayer’s hematoxylin (nuclear, purple) and eosin (cytoplasmic, pink) (H&E, original magnification, x 100 (left), x200 (right). Scare bar = 100 μm). Cryostat sections of normal-term placenta were stained with an antibody for Cytokeratin 7 (green) and PTTG1 (red). The sections were counterstained with DAPI. V, villi; magnification, ×400; scale bar = 50 μm. The bottom panels show higher magnification images of the regions indicated in the dotted line boxes. (C) Expression of PTTG1 mRNA in primary trophoblasts (PTB) isolated from normal-term placenta and immortalized human EVT cell line HTR-8/SVneo determined using qRT-PCR after 10 nM siPTTG1 treatment for 48 h. Values are means ± standard errors. *p < 0.05. (D) Expression of PTTG1 in primary trophoblasts (PTB) and HTR-8/SVneo cells analyzed by western blot after 10 nM siPTTG1 treatment for 48 h. β-actin or GAPDH was used as a loading control.

To analyze the expression of PTTG1 in normal-term placental tissues, qRT-PCR and western blotting were performed. Both PTTG1 mRNA and protein were expressed in placental tissues (Fig 1A). In addition, we analyzed the localization of PTTG1 in placental tissues using H&E staining and immunofluorescence. As shown on Fig 1B, histological sections of normal placental reveal a number of villi and several cluster of syncytiotrophoblasts sprouts to form syncytial knots. The expression of cytokeratin 7, which is a trophoblast-specific marker, was observed in a marginal region of villi in the placental tissues (e.g., syncytiotrophoblast), and the expression of PTTG1 was also found in trophoblasts in the outer layer of villi and capillary in placental tissues (Fig 1B). Thus, we confirmed the co-localization of PTTG1 and cytokeratin 7 in trophoblasts of placental villi. Furthermore, PTTG1 was expressed in PTBs and HTR-8/SVneo cells. The mRNA and protein expression levels of PTTG1 in both cell types were significantly decreased by siPTTG1 treatment (*p* < 0.05, Fig 1C and 1D).

### Effects of PTTG1 on proliferation of HTR-8/SVneo cells

Generally, PTTG1 regulates sister chromatid separation during mitosis and acts as a controlling factor for cell proliferation [17]. We performed MTT assay and FACS analysis to determine whether the effect of PTTG1 on HTR-8/SVneo cell cycling depended on the alteration of PTTG1 expression. There are no differences in proliferation and cell cycle of HTR-8/SVneo cells by siPTTG1 treatment (S1A and S1B Fig). Additionally, siPTTG1 treatment did not change the expression of survival- and cell cycle—related markers. However, knockdown of PTTG1 increased expression of phosphorylated Akt and Erk, and decreased p53 expression (*p* < 0.05, S1C Fig). These results indicate that the knockdown of PTTG1 by siPTTG1 did not affect HTR-8/SVneo cell proliferation.

### Decreased PTTG1 expression suppresses the invasion ability of HTR-8/SVneo cells by altering MMP-2 and MMP-9 expression

**Fig 2:**
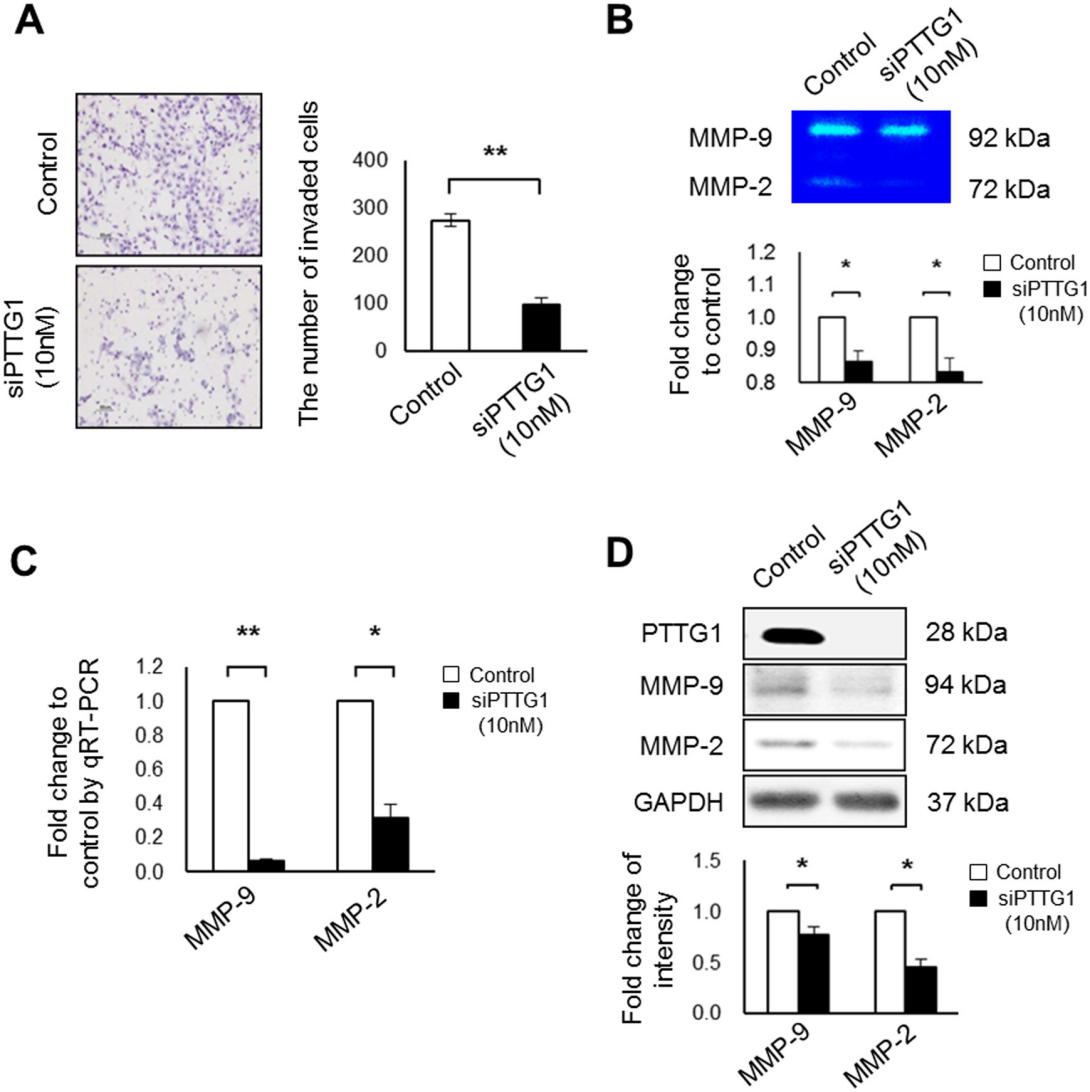
Invasion abilities of HTR-8/SVneo cells exposed to siPTTG1. (A) Images and numbers of invaded HTR-8/SVneo cells after siPTTG1 treatment (original magnification, ×100; scale bar = 80 μm). (B) Expression and densities of MMP-9 and MMP-2 in HTR-8/SVneo cells analyzed by zymography. (C) Expression of MMP-9 and MMP-2 mRNA in HTR-8/SVneo cells analyzed by qRT-PCR. (D) Expression and densities of MMP-9 and MMP-2 in HTR-8/SVneo cells after siPTTG1 treatment analyzed by western blot. Values are means ± standard errors. *p < 0.05 and **p < 0.001. β-actin or GAPDH was used as a loading control.

Since PTTG1 is involved in the invasion of lung cancer cells [21], we investigated whether PTTG1 induced the invasion ability of HTR-8/SVneo cells using Transwell chamber assays. The invasion ability of HTR-8/SVneo cells treated with siPTTG1 was significantly decreased compared with the controls (*p* < 0.001, Fig 2A). Furthermore, the expressions of MMP-2 and MMP-9 secreted from HTR-8/SVneo cells treated with siPTTG1 were decreased compared to the controls *p* < 0.05, Fig 2B). In addition, knockdown of PTTG1 in HTR-8/SVneo cells treated with siPTTG1 significantly reduced the expression of MMP-2 and MMP-9 at the mRNA and protein levels (*p* < 0.05, *p* < 0.001, Fig 2C and 2D). Similar to HTR-8/SVneo, the invasion ability of PTB cells treated with siPTTG1 was significantly decreased compared with the controls (*p* < 0.001, S2A Fig), and the expression of MMP-2 was regulated by siPTTG1 treatment (*p* < 0.05, S2B and S2C Fig). These findings suggest that knockdown of PTTG1 in trophoblast cells suppresses trophoblast cell invasion ability, as well as, expression of MMPs.

### Decreased PTTG1 expression suppresses the invasion ability of HTR-8/SVneo cells by altering the expression of adhesion molecules such as ITGA4, RhoA, and RhoC

**Fig 3:**
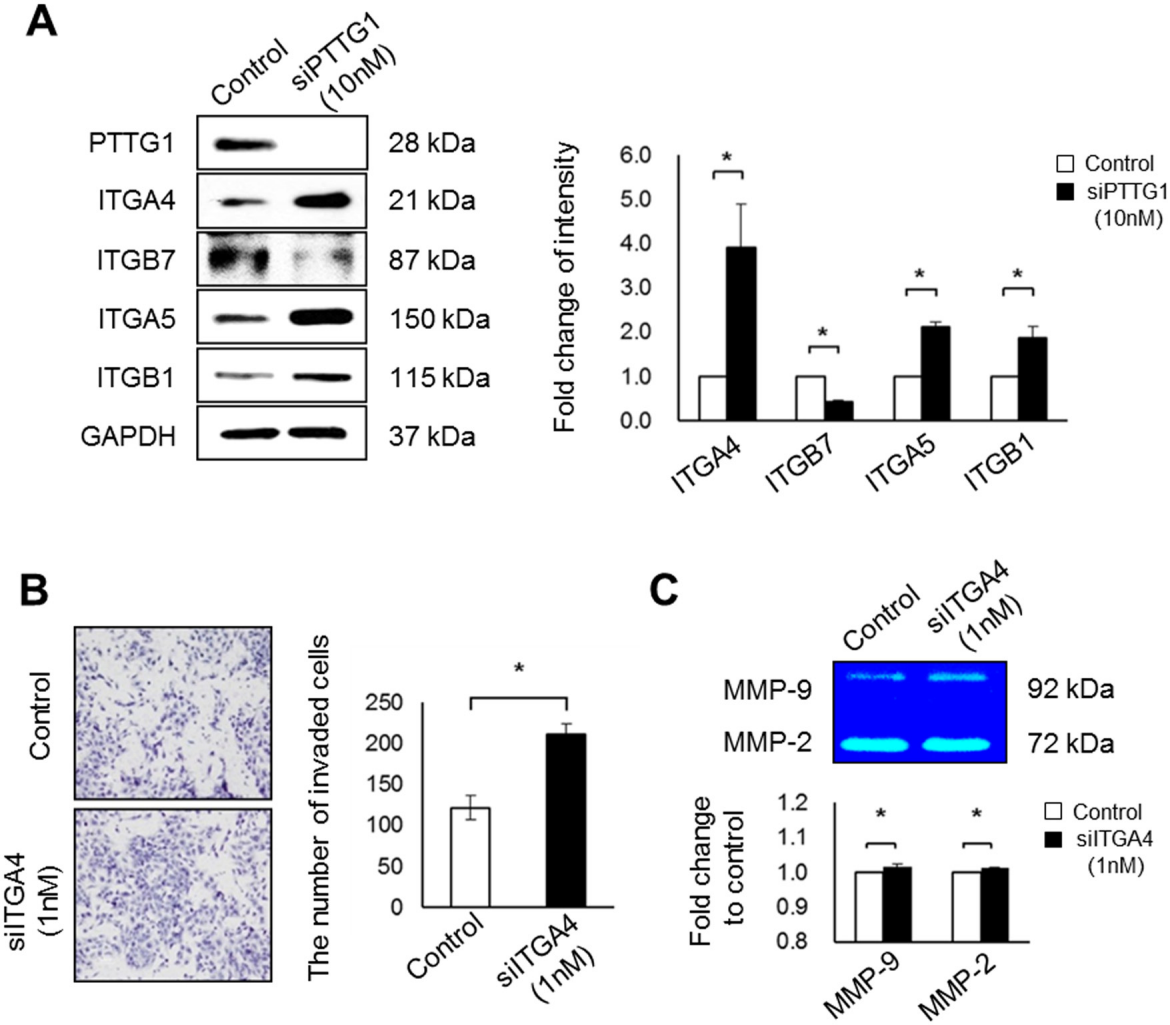
Expression of integrin family members and invasion abilities of HTR-8/SVneo cells treated with siITGA4. (A) Expression and densities of integrin family members in HTR-8/SVneo cells exposed to 10 nM siPTTG1, analyzed by western blot. (B) Images and numbers of invaded HTR-8/SVneo cells with 1 nM siITGA4 treatment for 48 h (original magnification, ×100; scale bar = 80 μm). (C) Expression of MMP-9 and MMP-2 in HTR-8/SVneo cells with siITGA4 treatment, analyzed by zymography. Values are means ± standard errors. *p < 0.05. GAPDH was used as a loading control.

Adhesion molecules (e.g., integrins) and Rho family members are important factors for cellular invasion [23]. In previous reports, we demonstrated that dynamic alterations of ITGA4 expression were involved in trophoblast invasion [7]. Thus, we investigated the relationship between integrins and PTTG1 in the invasion of HTR-8/SVneo cells. In cells treated with siPTTG1, the expression of integrins, including ITGA4, ITGA5, and integrin beta 1 (ITGB1), was significantly increased compared with the controls, whereas the expression of ITGB7 was decreased (*p* < 0.05, Fig 3A). Based on these results, we focused on the role of ITGA4 in the invasion ability of HTR-8/SVneo cells. The expression of ITGA4 was decreased by siITGA4 treatment in a dose- and time-dependent manner (*p* < 0.05, *p* < 0.001, S3A and S3B Fig). And the invasion ability of HTR-8/SVneo cells significantly increased when treated with siITGA4 (*p* < 0.05, Fig 3B). In addition, ITGA4 was down-regulated in HTR-8/SVneo cells treated with siITGA4, which enhanced the expression of MMP-2 and MMP-9, thereby increasing the invasion ability of trophoblast cells (*p* < 0.05, Fig 3C).

**Fig 4:**
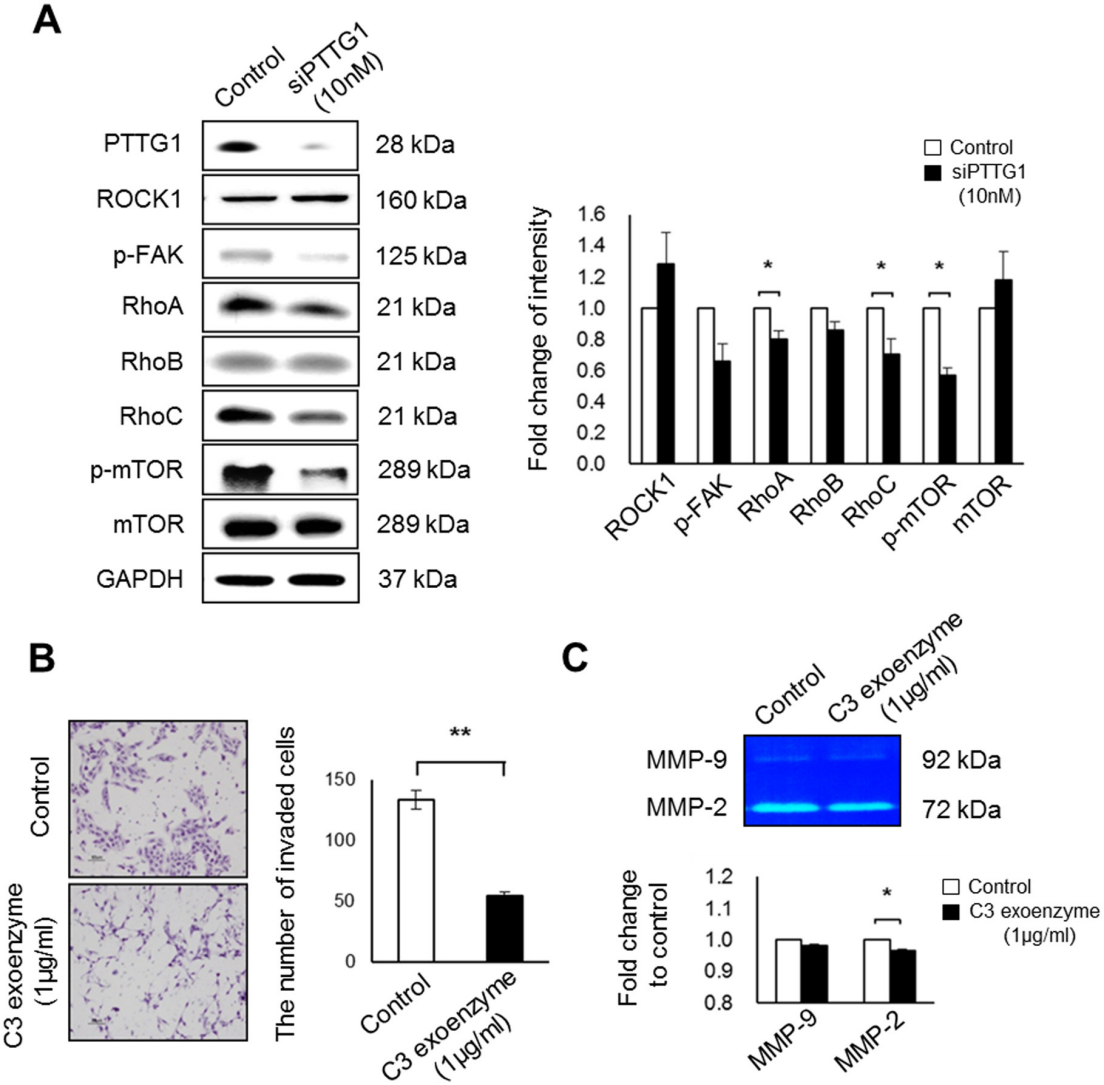
Expression of Rho family members and Invasion abilities of HTR-8/SVneo cells treated with C3 exoenzyme. (A) Expression and densities of Rho family members in HTR-8/SVneo cells treated with 10 nM siPTTG1, analyzed by western blot. (B) Image and numbers of invaded HTR-8/SVneo cells pre-treated with 1 μg/mL Rho inhibitor (C3 exoenzyme) for 3 h (original magnification, ×100; scale bar = 80 μm). (C) Expression of MMP-9 and MMP-2 in HTR-8/SVneo cells after Rho inhibitor treatment, analyzed by zymography. Values are means ± standard errors. *p < 0.05. **p < 0.001. GAPDH was used as a loading control.

The interaction between integrins and Rho-family GTPases, known as adhesion-dependent events in cells, regulates the invasion of various cancer cells [24]. Thus, we investigated whether Rho-family GTPases participated in the invasion of HTR-8/SVneo cells when regulated by PTTG1. Although the Rho kinase ROCK1 showed a tendency to increase in HTR-8/SVneo cells treated with siPTTG1, this expression was not significant (Fig 4A). The expression of RhoA and RhoC was significantly decreased in HTR-8/SVneo cells with siPTTG1 treatment, and this treatment reduced the phosphorylation of focal adhesion kinase and mammalian target of rapamycin (mTOR; *p* < 0.05, Fig 4A). To confirm the effects of Rho family members on the invasion of HTR-8/SVneo cells, we analyzed invasion and MMP expression in HTR-8/SVneo cells treated with C3 exoenzyme, an inhibitor of RhoA, RhoB, and RhoC. C3 exoenzyme treatment reduced the expression of Rho A/B/C, invasion ability of HTR-8/SVneo cells (*p* < 0.001, S3C Fig and Fig 4B), and the expression of MMP-2 (*p* < 0.05, Fig 4C). These data indicate that knockdown of PTTG1 decreased the invasion ability of HTR-8/SVneo cells through the down-regulation of RhoA and RhoC. The reduction of Rho family members by the C3 exoenzyme affected MMP-2, but not MMP-9, expression.

### miR-186-5p regulated invasion of HTR-8/SVneo cells via PTTG1 targeting

**Fig 5:**
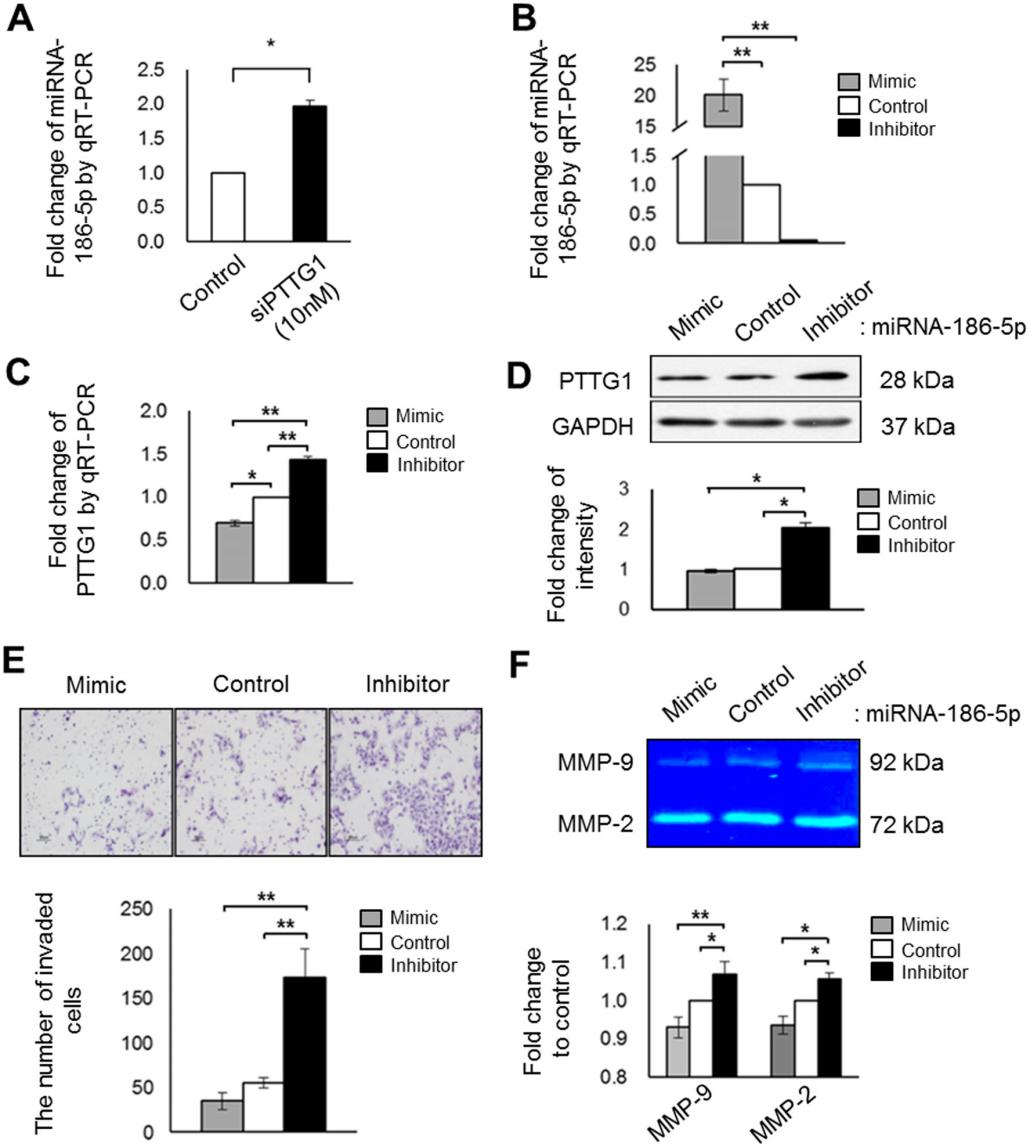
Correlation between PTTG1 and miRNA-186-5p in invasion ability of HTR-8/SVneo cells. (A) Expression of miRNA-186-5p in HTR-8/SVneo cells after 10 nM siPTTG1 treatment, analyzed by qRT-PCR. (B) Expression of miRNA-186-5p after miR-186-5p mimic or inhibitor treatment analyzed by qRT-PCR. (C) mRNA expression of PTTG1 in HTR-8/SVneo cells after treatment with 30 nM miR-186-5p. (D) Expression and density of PTTG1 in HTR-8/SVneo cells after treatment with 30 nM miR-186-5p. (E) Images and numbers of invaded HTR-8/SVneo cells exposed to 30 nM miR-186-5p (original magnification, ×100; scale bar = 80 μm). (F) Expression of MMP-9 and MMP-2 in HTR-8/SVneo cells after treatment with 30 nM miR-186-5p, analyzed by zymography. Values are means ± standard errors. *p < 0.05 and **p < 0.001. U6, β-actin, or GAPDH was used as a loading control.

PTTG1 regulates different invasion factors, such as ITGA4 and Rho family members, and their dynamic regulation may critically influence the invasion of HTR-8/SVneo cells. Thus, we hypothesized that a master regulator capable of controlling PTTG1 expression could regulate HTR-8/SVneo cell invasion by regulating the balances between different forms of invasion-related factors. To address this hypothesis, we identified several miRNA candidates predicted to bind to PTTG1 mRNA using bioinformatics analysis (microcosm: www.ebi.ac.uk/enright-srv/microcosm, TargetScan: www.targetscan.org, MicroRNA: www.microrna.org). We selected miRNA-186-5p, a candidate matching at least 7mer between miRNA and the mRNA 3’ UTR of PTTG1. We determined the expression of miR-186-5p in HTR-8/SVneo cells, regardless of PTTG1 expression. In qRT-PCR analysis, the expression of miR-186-5p was significantly increased in HTR-8/SVneo cells after siPTTG1 treatment (*p* < 0.05, Fig 5A). Additionally, the expressions of miRNA-186-5p expression by miR-186-5p mimic and inhibitor treatment were changed when PTTG1 expression in HTR-8/SVneo cells was decreased by siPTTG1 treatment, and the alterations are well matched with the patterns of miRNA-186-5p expression (*p* < 0.001, Fig 5B). Furthermore, miRNA-186-5p in cells treated with the mimic sequence showed increased expression levels of miRNA-186-5p, resulting in significantly decreased PTTG1 mRNA expression (*p* < 0.05, Fig 5C). In contrast, cells transfected with the miR-186-5p inhibitor sequence showed decreased expression levels of miRNA-186-5p, resulting in significantly increased PTTG1 mRNA expression compared with the negative controls (*p* < 0.001, Fig 5C). Similar results were observed in the mRNA data and protein expression levels of PTTG1 when regulated by miR-186-5p inhibitor although the expression of PTTG1 was not significant by miR-186-5p mimic treatment (*p* < 0.05 Fig 5D). These findings suggest that PTTG1 negatively miR-186-5p levels as well as miRNA-186-5p can control the expression of PTTG1 by targeting PTTG1 mRNA. Next, we determined whether miRNA-186-5p regulated the invasion of HTR-8/SVneo cells by altering PTTG1 expression. Using Transwell chamber assays and zymography, we confirmed that trophoblast cell invasion ability was inhibited by the miR-186-5p mimic sequence, and significantly enhanced by the miR-186-5p inhibitor sequence via alteration of MMP-2 and MMP-9 expression. But the proliferation of HTR-8/SVneo cells was not affected by miR-186-5p treatment (*p* < 0.05, *p* < 0.001, Fig 5E and 5F and S1A Fig). As well as, Increased invasion abilities of HTR-8/SVneo by miR-186-5p inhibitor were significantly reduced by combination with siPTTG1 ((*p* < 0.05, *p* < 0.001, S4A Fig).

These results suggest that miRNA-186-5p targeted PTTG1 mRNA and controlled the expression of PTTG1, resulting in the alteration of PTTG1 expression involved in the invasion ability of HTR-8/SVneo cells, by dynamically could be controlling RhoA and MMP expression.

## Discussion

During pregnancy, consistent support for fetal development through normal placental functions such as nutrient supply, gas exchange, and protection are necessary. Thus, normal placental development is important, and the trophoblast, which is derived from the blastocyst trophectoderm, has multiple functions and is critically involved in these processes. In particular, trophoblasts play roles in implantation and placentation due to their invasion activity, inducing implantation in early gestation and increasing blood flow through spiral artery remodeling in the maternal myoendometrium. If their invasion characteristics are manifested poorly or in an uncontrolled manner, the trophoblasts can retard fetal growth and induce several gynecological diseases such as preeclampsia (PE) and intrauterine growth restriction (IUGR) [25, 26]. For these reasons, studies of trophoblast function have used available trophoblast cell lines, representing the physiological conditions, since these lines are choriocarcinoma derived (e.g., JEG-3, BeWo, JAR) or immortalized by transformation of human extravillous trophoblast cells with the SV40 large T antigen (e.g., HTR-8/SVneo). Specifically, HTR-8/SVneo cells possess progenitor-like characteristics and are similar to primary trophoblasts [27, 28].

PTTG1, an oncogene, is involved in tumor progression, growth, proliferation, and metastasis by regulating several growth factors, such as fibroblast growth factor 2 and vascular endothelial growth factor [29]. Recently, PTTG1 was shown to regulate MMP-2 via direct promoter regulation, influence Rho-GEF ECT2, and be involved in the invasion and migration of breast cancer cells by promoting EMT [21, 30]. As well as, Shah et al., demonstrated that PTTG1 induce EMT in lung cancer though αVβ3-FAK and RhoA [31]. These evidence that PTTG1 regulates trophoblast invasion in a manner similar to the control of cancer metastasis. Because the correlation between PTTG1 and trophoblast invasion remains unclear, we demonstrated that PTTG1 expressed in trophoblasts, including primary trophoblasts (PTB), HTR-8/SVneo cell line, and placenta. Furthermore, PTTG1 was highly expressed in second trimester preeclampsia compared with normal-term placenta and third trimester preeclampsia (data not shown). Therefore, we hypothesized and demonstrated that trophoblast invasion ability could be regulated by alteration of PTTG1 at a point in the EMT. Based on our data, the expression of invasion-related EMT markers, including vimentin and cytokeratin peptide 18, did not change with siPTTG1 treatment (data not shown). Additionally, siPTTG1 did not change the morphology of trophoblasts. These findings suggest that decreased PTTG1 is not involved in characteristic trophoblast alteration. PTTG1 regulates the invasion ability of HTR-8/SVneo cells by increasing the expression of MMP-2 and MMP-9 via abundant expression in the cytoplasm of invasive HTR-8/SVneo cells (data not shown). These findings suggest that PTTG1 may play important roles in the invasion of HTR cells via regulation of MMPs, but these pathways differ from those of malignant tumors (e.g., choriocarcinoma) [32, 33].

MMPs are not only necessary for development, but are also associated with cancer metastasis and poor prognosis [34, 35]. MMP activation is mediated by several microenvironmental factors, including adhesion molecules (e.g., integrins) and Rho family members [36]. Among MMPs, the gelatinases MMP-2 and MMP-9 promote trophoblast invasion by degrading collagen type IV in the endometrium [10]. In the present study, we showed that suppression of ITGA4 or RhoA/B/C affects trophoblast invasion and alters MMP-2 and MMP-9 expression. Additionally, decreased PTTG1 and ITGA4 suppressed MMP-2 and MMP-9 expression, but inhibition of RhoA/B/C by C3 exoenzyme reduced only MMP-2 expression. These different mechanisms should be studied further because PTTG1 is involved in the regulation of trophoblast invasion via alteration of integrins and Rho family members. In addition, we analyzed the expression of PTTG1 after siMMP-2 and siMMP-9 treatment to evaluate interaction between PTTG1 and MMPs. We confirmed that the expressions of PTTG1 were significantly reduced by siMMP-2 and siMMP-9 treatment (S4B and S4C Fig). However, the further studies should be needed to evaluate the correlationship between PTTG1 and MMPs to migration of trophoblast cells.

Integrins are adhesion receptors composed of alpha and beta subunits, and their heterodimer complexes mediate cell—cell- and cell—extracellular matrix-related adhesion processes by recognizing their ligands, including extracellular matrix proteins, growth factors, and cytokines [37]. In addition, integrins play important roles in cancer and trophoblast invasion through interaction between integrins and Rho GTPase [38, 39]. Rho GTPase families are molecular switches that regulate many essential cellular processes, including actin dynamics, gene transcription, cell cycle progression, and cell adhesion [40]. Moreover, Rho GTPases are involved in cancer invasion, metastasis, and trophoblast invasion [8, 41]. In the present study, we showed that decreased PTTG1 significantly suppressed the expression of RhoA and RhoC and the phosphorylation of mTOR. The invasion ability of HTR-8/SVneo cells was suppressed by C3 exoenzyme, a specific RhoA/B/C inhibitor, similar to previously reported results [8, 42].

This study is the first to show that PTTG1 knockdown altered the expression of integrin family members, including ITGA4, ITGB7, ITGA5, ITGB1, and Rho family members. Specifically, siPTTG1 significantly increased the expression of ITGA4 in HTR-8/SVneo cells. These findings are in agreement with our previous report showing that the alteration of ITGA4 by hypoxia negatively regulated the invasion ability of HTR-8/SVneo cells [7]. Conversely, Spessotto et al. [43] reported that ITGA4 induced trophoblast invasion. Thus, we focused on ITGA4 expression and confirmed its effect on invasion ability and MMP secretion after siITGA4 treatment. The data verified that ITGA4 is a negative regulator of HTR-8/SVneo cell invasion. Furthermore, expression of PTTG1 increased in cultured cells under 0.1% hypoxia for 24 h (data not shown). The difference in results can be explained by the correlation between gene function and environmental factors.

Recent studies have also suggested that miRNAs are involved in many cellular processes through the post-translational regulation of several genes. Thus, the mining and evaluation of specific miRNA-targeted genes involved in various diseases is a possible gene therapy method [44]. Down-regulation of miRNA-186-5p was associated with poor survival in lung adenocarcinoma through the targeting of cyclin D1 and cyclin-dependent kinases 2 and 6 [45]. In addition, miRNA-186, -216b, -337-3p, and -760 were shown to be involved in senescence by targeting the CK2 alpha subunit [46]. Moreover, the natural product curcumin regulated apoptosis in lung adenocarcinoma cells by suppressing miRNA-186-5p [47]. Using bioinformatics algorithms, including microRNA [48], TargetScan Human [49], and Microcosm Targets [50], we identified miRNA-186-5p, which targets PTTG1 and was presumed to be important in HTR-8/SVneo cell invasion. siPTTG1 significantly increased the expression of miRNA-186-5p, indicating that miRNA-186-5p controls PTTG1 expression. The invasion ability of HTR-8/SVneo cells was regulated by PTTG1-targeting miRNA-186-5p, suggesting that miRNA-186-5p could be a regulator of invasion of HTR-8/SVneo cell invasion through regulation of PTTG1 expression, and may have the potential to be a diagnostic marker in invasion-related diseases.

In conclusion, this study is the first to show that PTTG1 is an important factor in the regulation of trophoblast invasion, and alteration in its expression induces dynamic changes in the expression of ITGA4 and Rho family members. Furthermore, miRNA-186-5p targets PTTG1 and regulates its expression. Therefore, these findings contribute to the understanding of the implantation mechanism and placental development by highlighting the role of PTTG1 in trophoblast invasion. In addition, our results could be used not only for therapies targeting trophoblasts in invasion-related diseases with poor prognosis, but also in therapies for pregnancy-associated diseases. However, further studies and the identification of other products targeted by miRNA-186-5p should be necessary.

## Supporting Information

S1 Table Antibody used in the study. (DOC)

S1 Fig Proliferation of HTR-8/SVneo cells treated with siPTTG1 or miR-186-5p. (A) Viabilities of HTR-8/SVneo cells after 10nM siPTTG1 for 48 h (Left), and miR-186-5p-targeting mimic or inhibitor treatment for 24 h (Right) using MTT assay. (B) Cell cycle of HTR-8/SVneo cells after 10 nM siRNA treatment for 48 h, analyzed by FACS. (C) Expression and density of cell cycle—related factors in HTR-8/SVneo cells after 10 nM siPTTG1 treatment for 48 h. Values are means ± standard errors. *p < 0.05. GAPDH was used as a loading control.
(TIF)

S2 Fig Invasion abilities of PTB cells exposed to siPTTG1 treatment. (A) Images and numbers of invaded PTB cells after siPTTG1 treatment (original magnification, ×100; scale bar = 80 μm). (B) Expression and densities of MMP-9 and MMP-2 in PTB cells analyzed by zymography. (C) Expression of MMP-9 and MMP-2 mRNA in PTB cells, analyzed by qRT-PCR. Values are means ± standard errors. *p < 0.05 and **p < 0.001. β-actin was used as a loading control.
(TIF)

S3 Fig Expression of ITGA4 and Rho A/B/C on HTR-8/SVneo cells treated with siITGA4 and Rho inhibitor (C3 exoenzyme). (A) mRNA expression of ITGA4 in HTR-8/SVneo cells treated with siITGA4 treatment, analyzed by qRT-PCR. Values are means ± standard errors. *p < 0.05 and **p < 0.001. (B) Expression of ITGA4 in HTR-8/SVneo cells after 0, 1, and 10 nM siITGA4 treatment for 24 or 48 h, analyzed by western blot. (C) Expression of Rho A/B/C in HTR-8/SVneo cells after 1 μg/mL C3 exoenzyme treatment for 3 h, analyzed by western blot. GAPDH was used as a loading control.
(TIF)

S4 Fig Invasion abilities of HTR-8/SVneo cells by miR-185-5p inhibitor and expressions of PTTG1 on HTR-8/SVneo cells depend on MMPs expression. (A) Images and numbers of invaded HTR-8/SVneo cells after treatment of 30nM miR-186-5p mimic or inhibitor, and combination of miR-186-5p inhibitor and 10nM siPTTG1 (original magnification, ×100; scale bar = 80 μm). (B) Expressions and (C) intensities of PTTG1 in HTR-8/SVneo cells after siRNAs treatment including 10nM siPTTG1, 50nM siMMP-2, and 50nM siMMP-9 for 48 h using Western blot. GAPDH was used as a loading control. Values are means ± standard errors. *p < 0.05 and **p < 0.001.
(TIF)

## Abbreviations

PTTG1: Pituitary tumor transforming gene-1
PTB: primary trophoblast
CTB: Cytotrophoblast
MMP: matrix metalloproteinase
ITGA: integrin alpha
ITGB: integrin beta
GTPases: small guanosine triphosphatases
ROCK1: Rho-associated, coiled-coil containing protein kinase1
FAK: Focal adhesion kinase
mTOR: Mammalian target of rapamycin
STAT3: Signal transducer and activator of transcription
PRL-3: Phosphatase of regenerating liver-3
Erk: Extracellular signal-regulated kinases
EMT: Epithelial-mesenchymal transition
PE: preeclampsia
IUGR: intrauterine growth restriction
FACS: fluorescence-activated cell sorting
siRNA: small interfering RNA
miRNA: microRNA
SV40: simian virus40
Tag: large T antigen
FGF4: fibroblast growth factor
