# Supplementary material for: Alteration of Pituitary Tumor Transforming Gene-1 Regulates Trophoblast Invasion via the Integrin/Rho-Family Signaling Pathway
Source: PLoS One. 2016 Feb 22;11(2):e0149371. doi: 10.1371/journal.pone.0149371 (PMC4764760; doi:10.1371/journal.pone.0149371)
Supplement: S1 Table — (DOC) [file pone.0149371.s001.doc]

S1 Table. Antibody used in the study.

| **Antibody** | **Sources** | **Dilution factor** | **Catalog number** | **Company** |
| --- | --- | --- | --- | --- |
| **PTTG1** | Rabbit | 1 : 1000 | 34-1500 | invitrogen |
| **Cyclin A** | Rabbit | 1 : 1000 | Ab7956 | Abcam |
| **Cyclin E** | Rabbit | 1 : 1000 | 630701 | Biolegend |
| **p-STAT3** | Rabbit | 1 : 1000 | 9134,9131 | Cell signaling |
| **STAT3** | Rabbit | 1 : 1000 | Sc-483 | Santa Cruz |
| **p-AKT** | Rabbit | 1 : 1000 | #9271 | Cell signaling |
| **AKT1** | Mouse | 1 : 1000 | Sc-5298 | Santa Cruz |
| **p-Erk** | Mouse | 1 : 1000 | 9106 | Cell signaling |
| **P53** | Mouse | 1 : 1000 | Sc-126 | Santa Cruz |
| **MMP-9** | Rabbit | 1 : 1000 | Ab76003 | Abcam |
| **MMP-2** | Rabbit | 1 : 1000 | Ab79781 | Abcam |
| **Integrin alpha4** | Rabbit | 1 : 500 | 4827 | proSci |
| **Integrin beta7** | Rabbit | 1 : 1000 | Ab108926 | abcam |
| **Integrin alpha5** | Mouse | 1 : 1000 | 610634 | BD biosciences |
| **Integrin beta1** | Mouse | 1 : 1000 | MAB1778 | R&D systems |
| **ROCK1** | Rabbit | 1 : 1000 | 4035 | Cell signaling |
| **p-FAK** | Rabbit | 1 : 1000 | 3283 | Cell signaling |
| **Rho A** | Rabbit | 1 : 1000 | 2117 | Cell signaling |
| **Rho B** | Rabbit | 1 : 1000 | 2098 | Cell signaling |
| **Rho C** | Rabbit | 1 : 1000 | 3430 | Cell signaling |
| **p-mTOR** | Rabbit | 1 : 1000 | 5536 | Cell signaling |
| **mTOR** | Rabbit | 1 : 1000 | 2983 | Cell signaling |
| **GAPDH** | Rabbit | 1 : 3000 | LF-PA0018 | Abfrontier |
