# Supplementary figures and images for: Alteration of Pituitary Tumor Transforming Gene-1 Regulates Trophoblast Invasion via the Integrin/Rho-Family Signaling Pathway
Source: PLoS One. 2016 Feb 22;11(2):e0149371. doi: 10.1371/journal.pone.0149371 (PMC4764760; doi:10.1371/journal.pone.0149371)

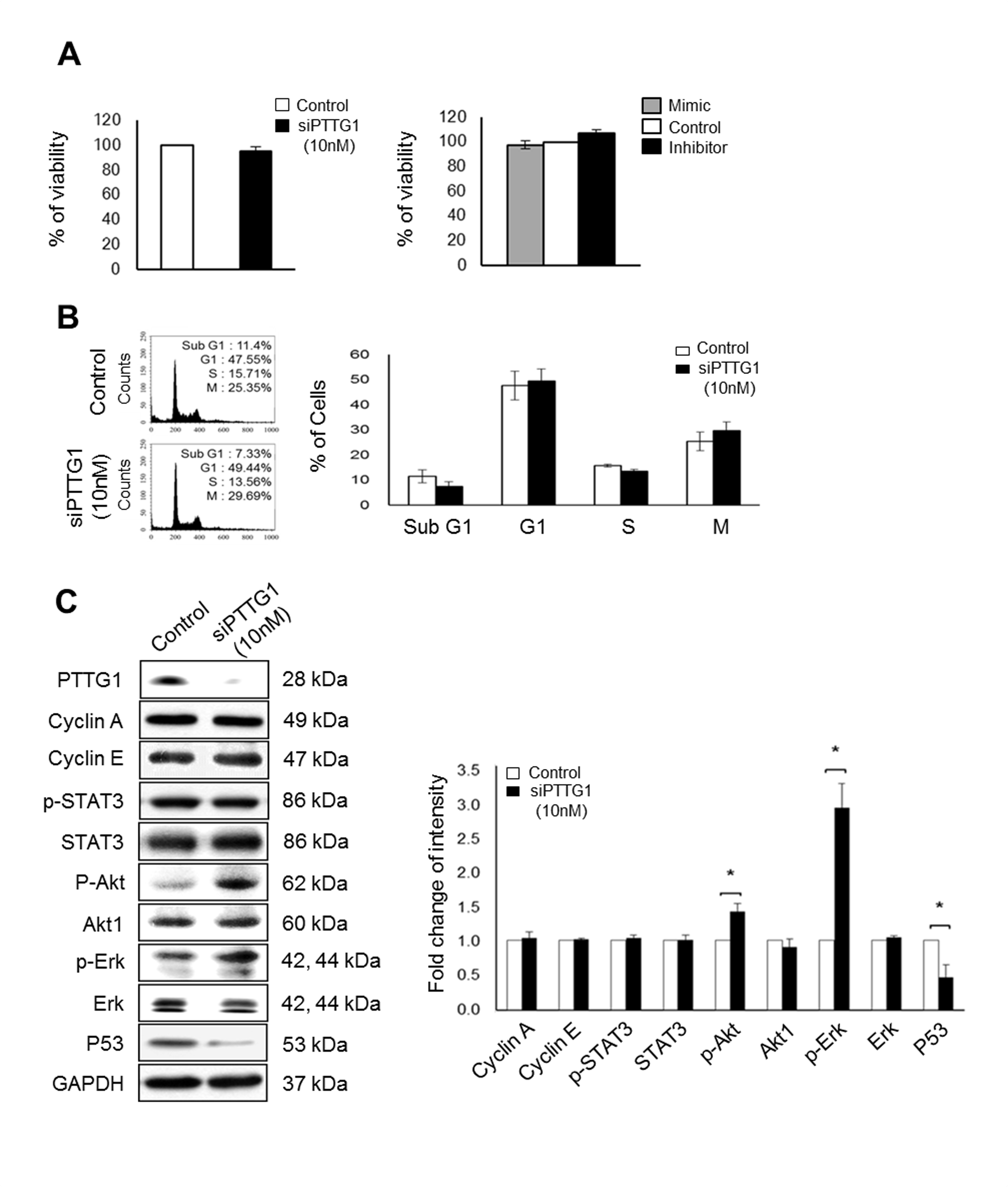

Supplement: S1 Fig — (A) Viabilities of HTR-8/SVneo cells after 10nM siPTTG1 for 48 h (Left), and miR-186-5p-targeting mimic or inhibitor treatment for 24 h (Right) using MTT assay. (B) Cell cycle of HTR-8/SVneo cells after 10 nM siRNA treatment for 48 h, analyzed by FACS. (C) Expression and density of cell cycle—related factors in HTR-8/SVneo cells after 10 nM siPTTG1 treatment for 48 h. Values are means ± standard errors. *p < 0.05. GAPDH was used as a loading control. (TIF) [file pone.0149371.s002.tif]

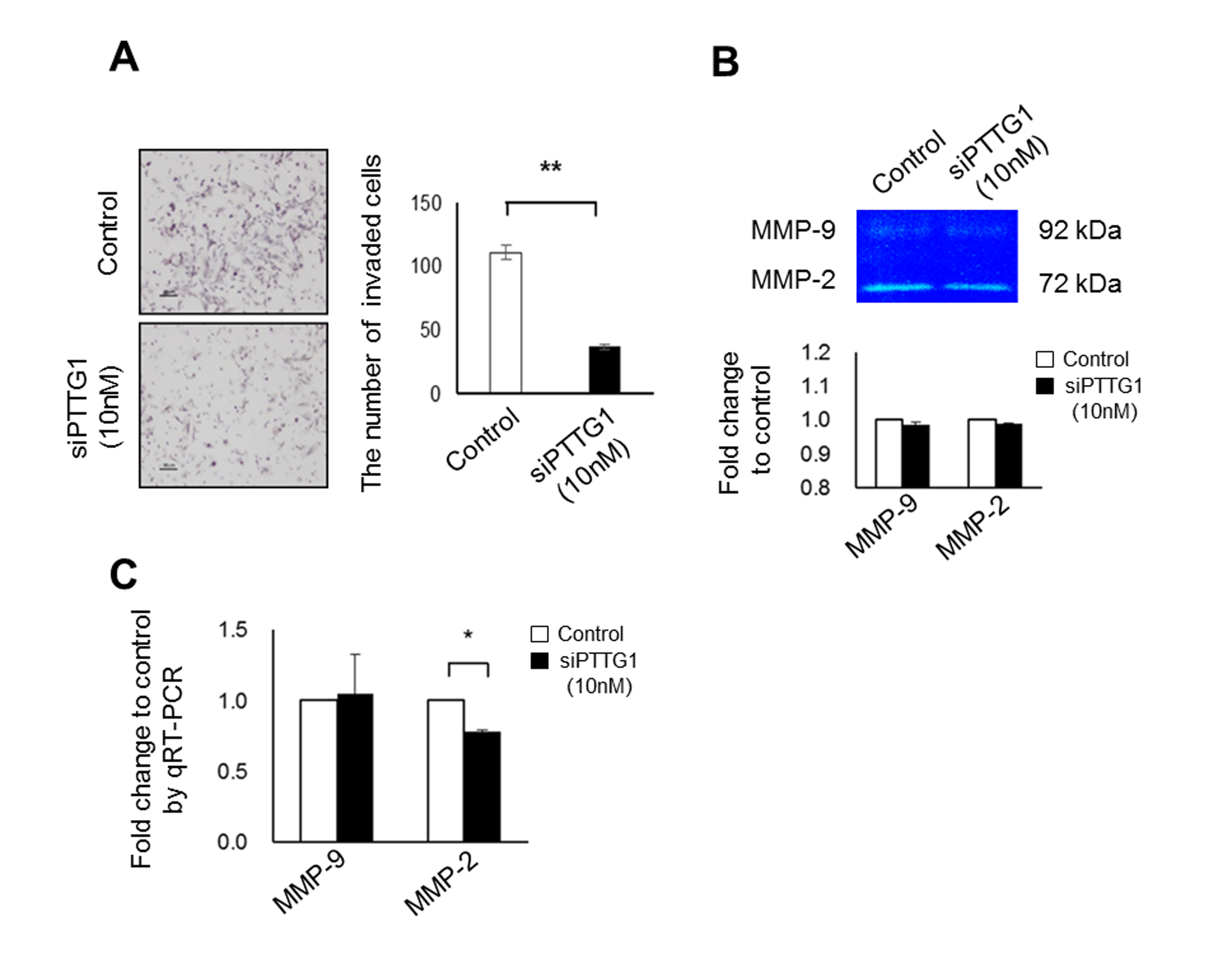

Supplement: S2 Fig — (A) Images and numbers of invaded PTB cells after siPTTG1 treatment (original magnification, ×100; scale bar = 80 μm). (B) Expression and densities of MMP-9 and MMP-2 in PTB cells analyzed by zymography. (C) Expression of MMP-9 and MMP-2 mRNA in PTB cells, analyzed by qRT-PCR. Values are means ± standard errors. *p < 0.05 and **p < 0.001. β-actin was used as a loading control. (TIF) [file pone.0149371.s003.tif]

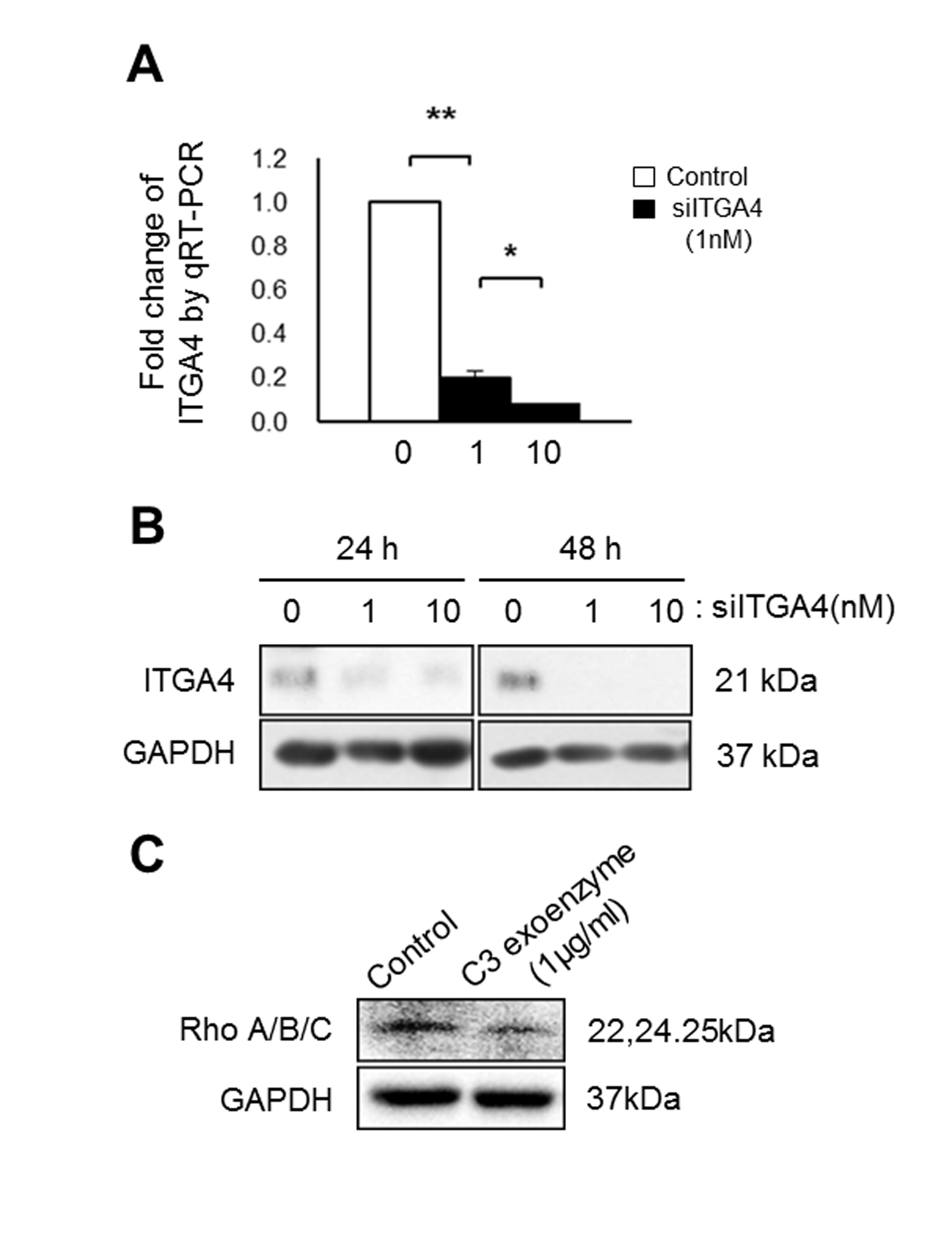

Supplement: S3 Fig — (A) mRNA expression of ITGA4 in HTR-8/SVneo cells treated with siITGA4 treatment, analyzed by qRT-PCR. Values are means ± standard errors. *p < 0.05 and **p < 0.001. (B) Expression of ITGA4 in HTR-8/SVneo cells after 0, 1, and 10 nM siITGA4 treatment for 24 or 48 h, analyzed by western blot. (C) Expression of Rho A/B/C in HTR-8/SVneo cells after 1 μg/mL C3 exoenzyme treatment for 3 h, analyzed by western blot. GAPDH was used as a loading control. (TIF) [file pone.0149371.s004.tif]

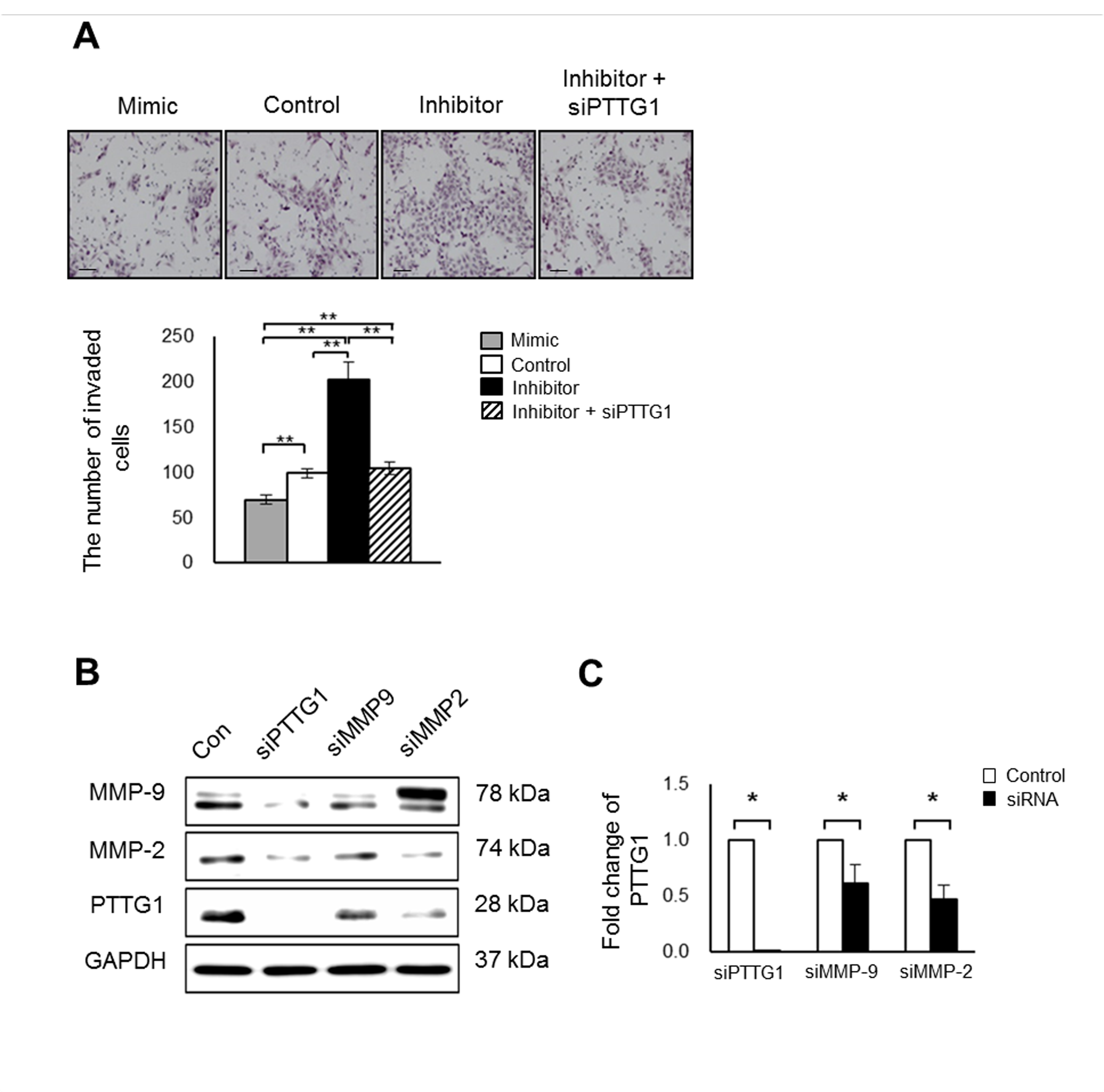

Supplement: S4 Fig — (A) Images and numbers of invaded HTR-8/SVneo cells after treatment of 30nM miR-186-5p mimic or inhibitor, and combination of miR-186-5p inhibitor and 10nM siPTTG1 (original magnification, ×100; scale bar = 80 μm). (B) Expressions and (C) intensities of PTTG1 in HTR-8/SVneo cells after siRNAs treatment including 10nM siPTTG1, 50nM siMMP-2, and 50nM siMMP-9 for 48 h using Western blot. GAPDH was used as a loading control. Values are means ± standard errors. *p < 0.05 and **p < 0.001. (TIF) [file pone.0149371.s005.tif]
